# Cure of Aneurism by Insertion of Wire

**Published:** 1885-07

**Authors:** 


### CURE OF ANEURISM BY INSERTION OF WIRE.

Says the New Orleans *Medical and Surgical Journal* for July: "Another wonderful triumph has been added to the surgeons' crown of laurels.

"'Signor Loreta, of Bologna, has cured an abdominal aneurism by cutting down upon it, and inserting into the sac two metres of silvered copper wire. The first night the patient rested free from the acute pain, which he had been enduring for months, the femoral pulse, until then barely perceptible, gradually returned, and he went on to complete recovery, being discharged three weeks after the operation."

"This practice is probably only adapted to sacular aneurisms, and great care must be observed to prevent the wire from adhering to the inner sides of the tumor."

"Our iodide of potassium, sugar of lead, ergot, ice compresses, etc., will no longer be needed, if the surgeon can thus come to the aid of the physician."

Aid? It looks like relieving him from duty altogether. Say, "supercede the physician."

